# Supplementary material for: The origin of magnetization-caused increment in water oxidation
Source: Nat Commun. 2023 Apr 29;14:2482. doi: 10.1038/s41467-023-38212-2 (PMC10148796; doi:10.1038/s41467-023-38212-2)
Supplement: Supplementary file 1 — Supplementary Information [file 41467_2023_38212_MOESM1_ESM.pdf]

Supplementary materials for: The origin of magnetization caused increment in water oxidation

Xiao Ren[1,2,3,7], Tianze Wu[1,7], Zizhao Gong[1,2], Lulu Pan[2], Jianling Meng[4], Haitao Yang[2], Freyja Bjork Dagbjartsdottir[5], Adrian Fisher[5], Hong-Jun Gao[2], Zhichuan J. Xu[1,6] *

[1] School of Material Science and Engineering, Nanyang Technological University, 50 Nanyang Avenue, Singapore 639798, Singapore;

[2] Beijing National Laboratory for Condensed Matter Physics and Institute of Physics, Chinese Academy of Science, P.O.Box 603, Beijing, 100190, China;

[3] Beijing National Laboratory for Molecular Engineering, College of Chemistry and Molecular Engineering, Peking University, Beijing 100871, China;

[4] College of Mathematics and Physics, Beijing University of Chemical Technology, Beijing, 100029, China;

[5] Department of Chemical Engineering, University of Cambridge, Cambridge CB2 3RA, UK;

[6] Energy Research Institute @ Nanyang Technological University, 50 Nanyang Avenue, Singapore 639798, Singapore.

[7] These authors contributed equally.

E-mails: xuzc@ntu.edu.sg

Contents:

Supplementary figure

The schematic diagram of the working electrode preparation

The EDX mapping and element analysis of NiFe films with different thicknesses (Fig.2-3)

The EDX mapping and element analysis of the surface region of NiFe films (Fig.4-5)

Equilibrium magnetization distribution obtained from micromagnetic simulation (Fig.6)

The MFM images from micromagnetic simulation (Fig.7)

Domain wall areas and their calculation methods (Fig.8)

Normalized remanent magnetization and saturation field of NiFe films details (Fig.9)

Pretreatment (5 CVs) for NiFe films (Fig.10)

The XPS analysis of NiFe films with and without electrochemical treatment (Fig.11-12)

The magnetic hysteresis loops of NiFe film after OER treatment (Fig.13)

The TEM images of NiFe film after OER treatment (Fig.14)

Cycling (200 CVs) for NiFe films (Fig.15)

CVs for NiFe films with and without magnetic field after pretreatment (100 CVs) (Fig.16)

Tafel, EIS and double layer for NiFe films with and without magnetic field (Fig.17-25)

MFM images for initial sample, after pre-treatment sample and after OER sample (Fig.26)

The relationship between OER enhancement and the ratio of domain wall (Fig.27)

Supplementary Table

Film thicknesses and atom ratio obtained from SEM and EDX

Surface analysis of NiFe thin films

References

Supplementary discussion

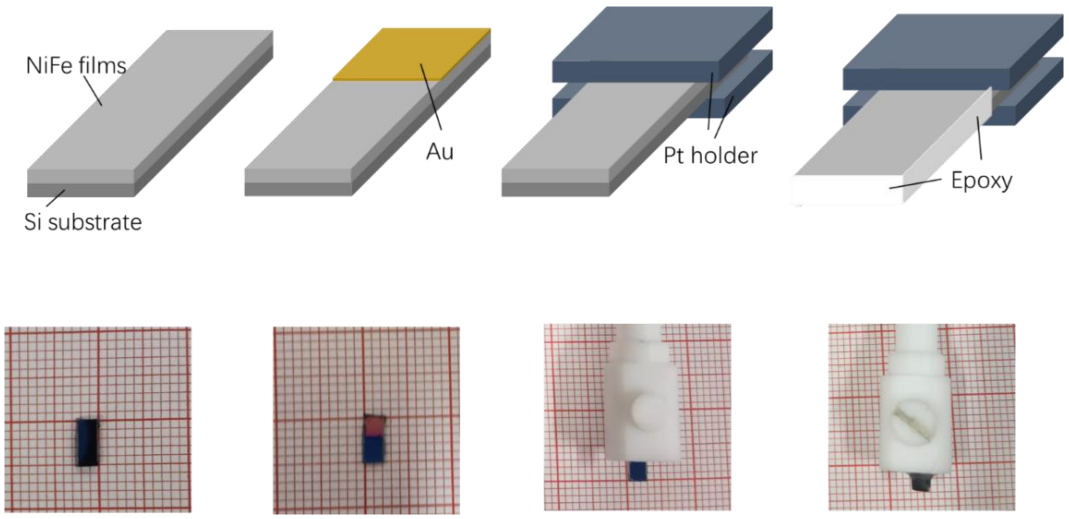

Supplementary Fig. 1. The schematic diagram of working electrode preparation.

47

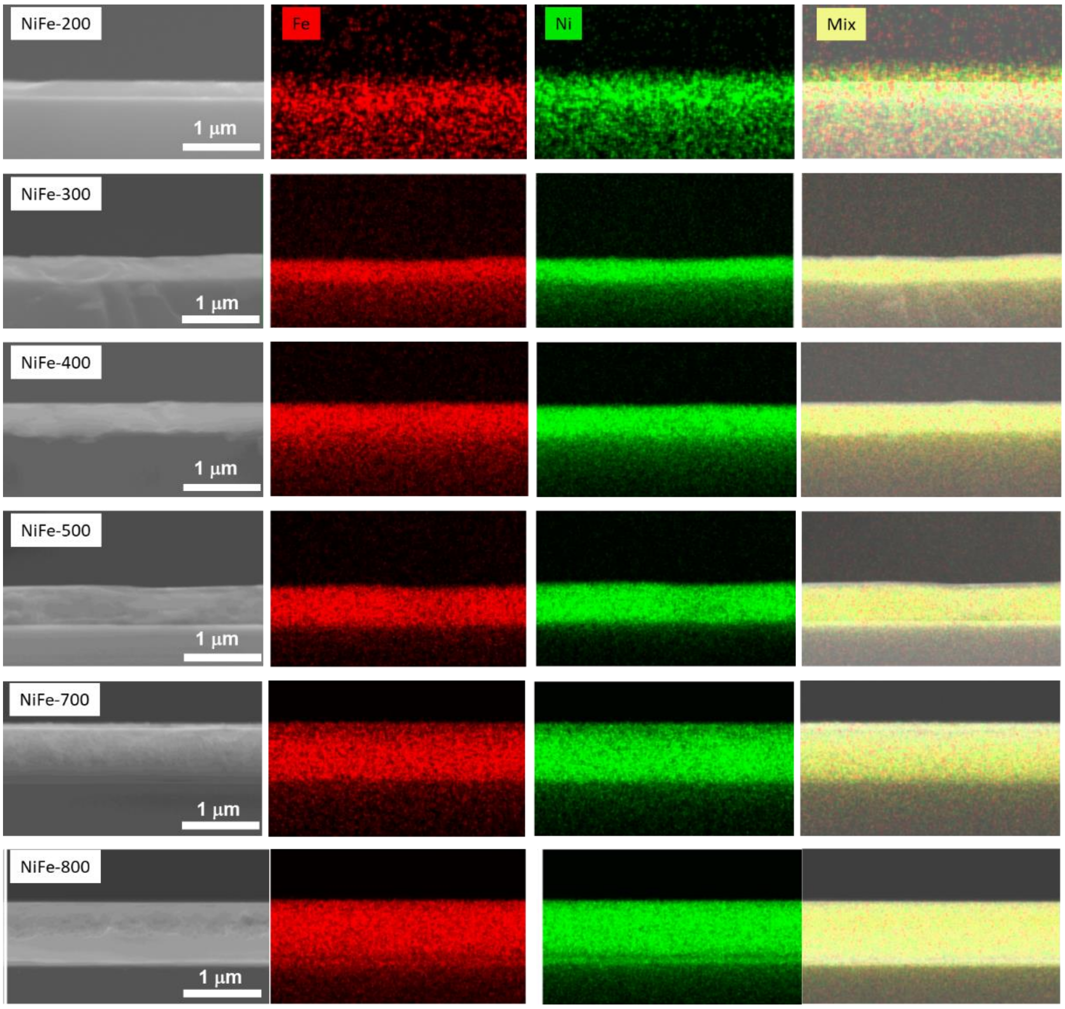

48 **Supplementary Fig. 2.** The EDX mapping of NiFe films with different thicknesses.

49

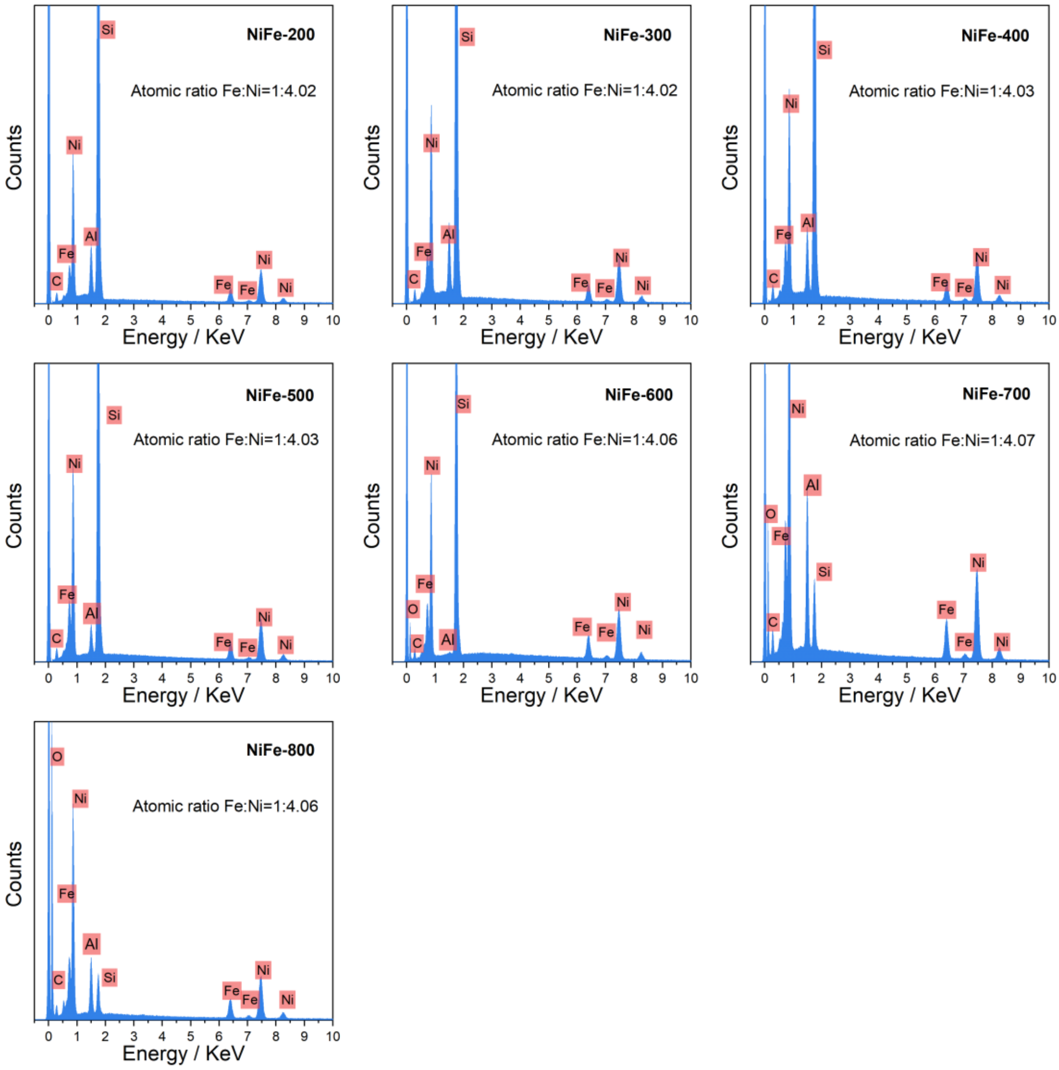

Supplementary Fig. 3. | The element analysis by using the energy-dispersive x-ray spectroscopy (EDX). The EDX are measured in multiple regions of each NiFe film. The chemical composition of Fe:Ni of all NiFe films is ~ 1:4 and summarized in Supplementary Table 1.

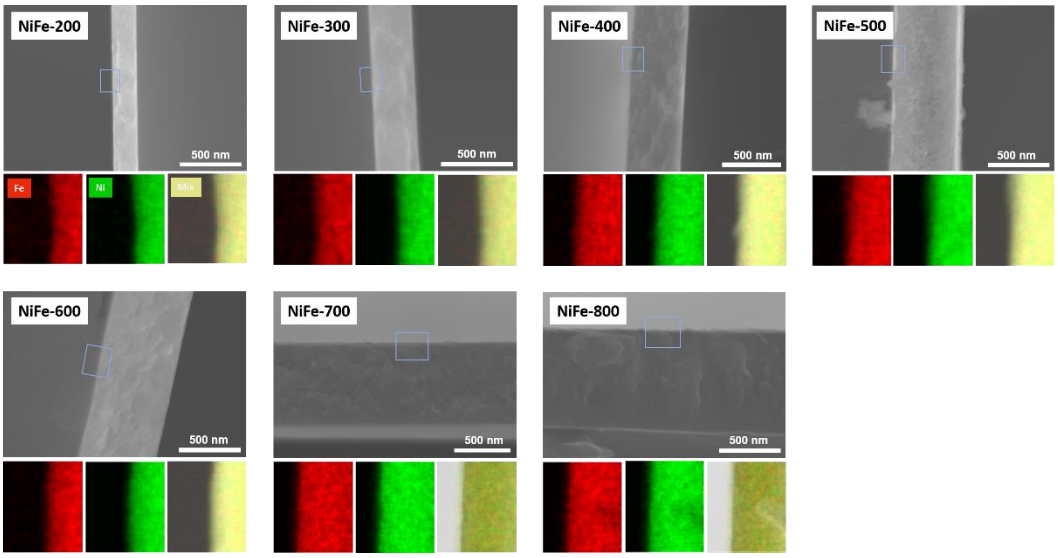

Supplementary Fig. 4. The EDX mapping of the surface region of NiFe films with different thicknesses.

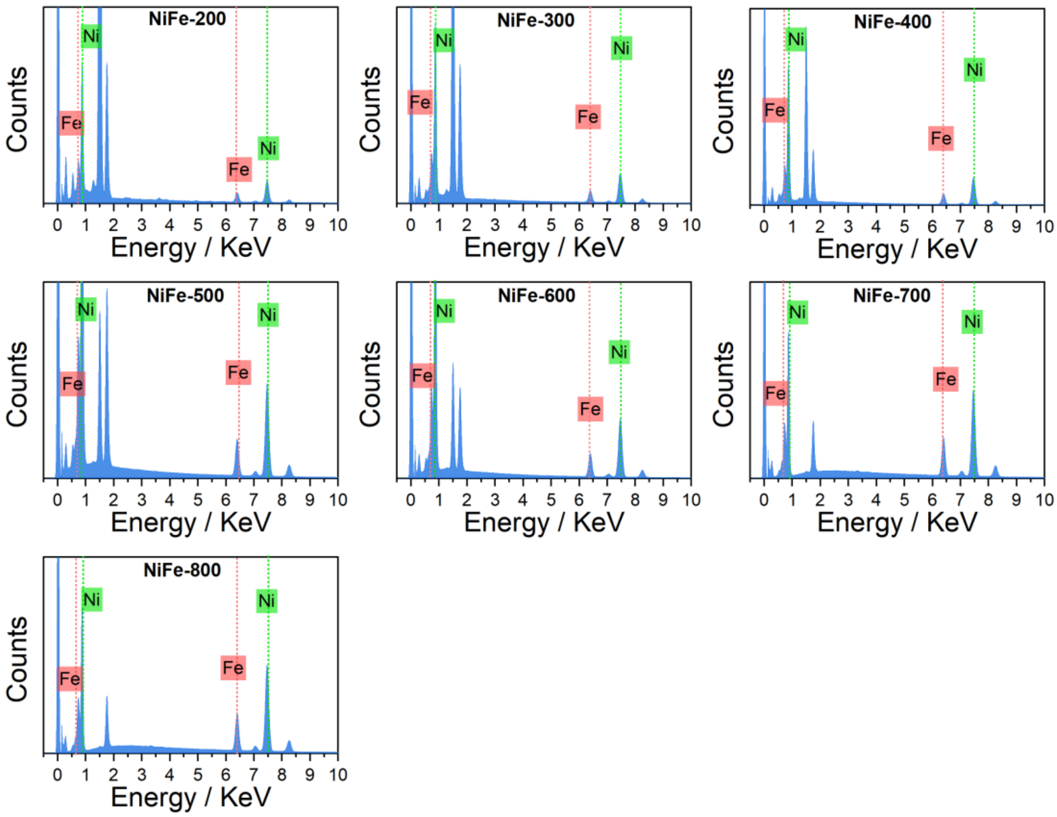

Supplementary Fig. 5. The representative EDX spectra of NiFe films. Each film is measured for three times at different regions. The measured atomic ratios (Ni:Fe) are summarized in Supplementary Table 3.

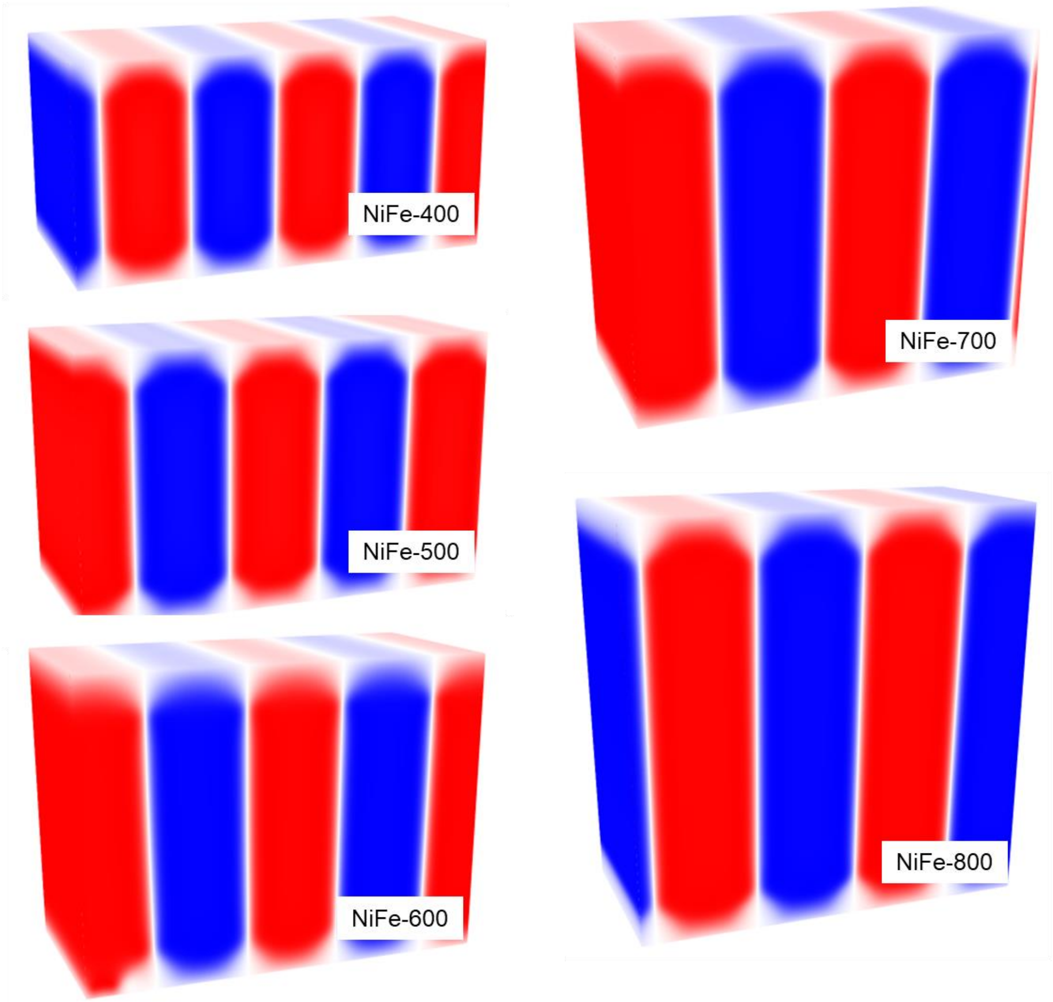

Supplementary Fig. 6. Equilibrium magnetization distribution obtained from micromagnetic simulation of NiFe films with different thicknesses.

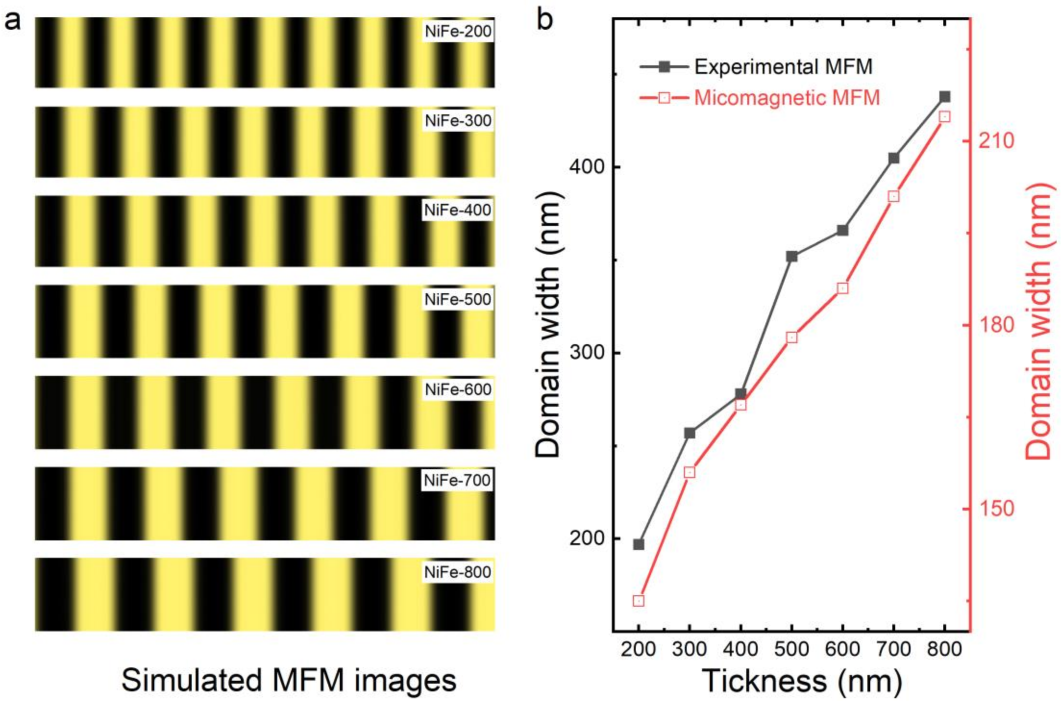

Supplementary Figure 7. a. The MFM images from micromagnetic simulation; b, Domain width of the NiFe thin films with the different thickness obtained from experimental MFM and micromagnetic MFM.

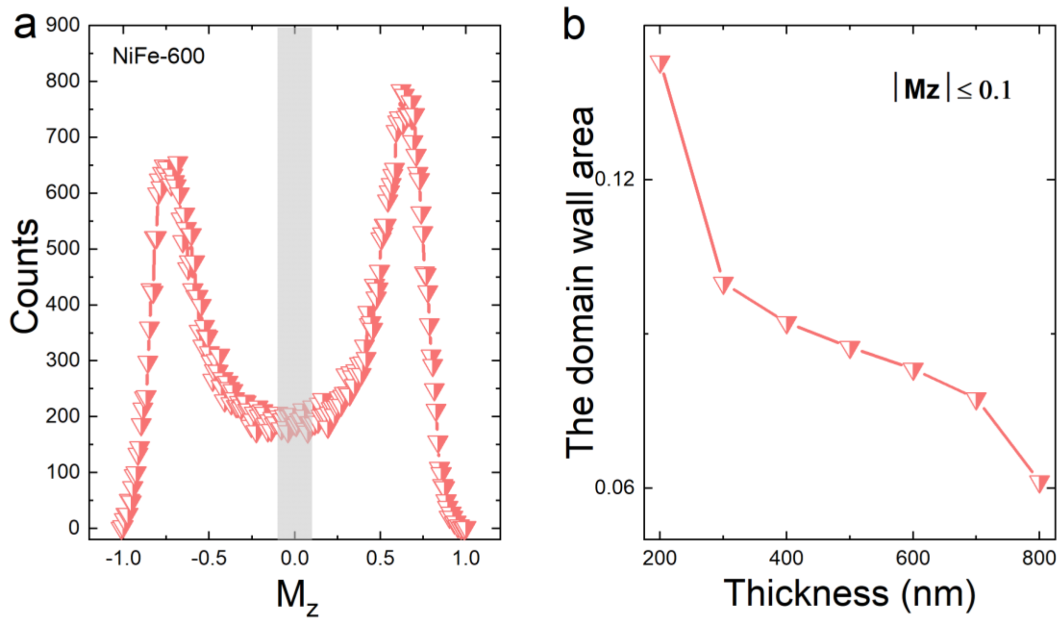

Supplementary Fig. 8. | Domain wall areas and their calculation methods. a. The counts in MFM images (exemplified with NiFe-600) as a function of Mz. The domain wall proportion is obtained by the ratio of the counts with |Mz| ≤ 0.1 to the total counts. b. Domain wall areas of the NiFe thin films with different thicknesses.

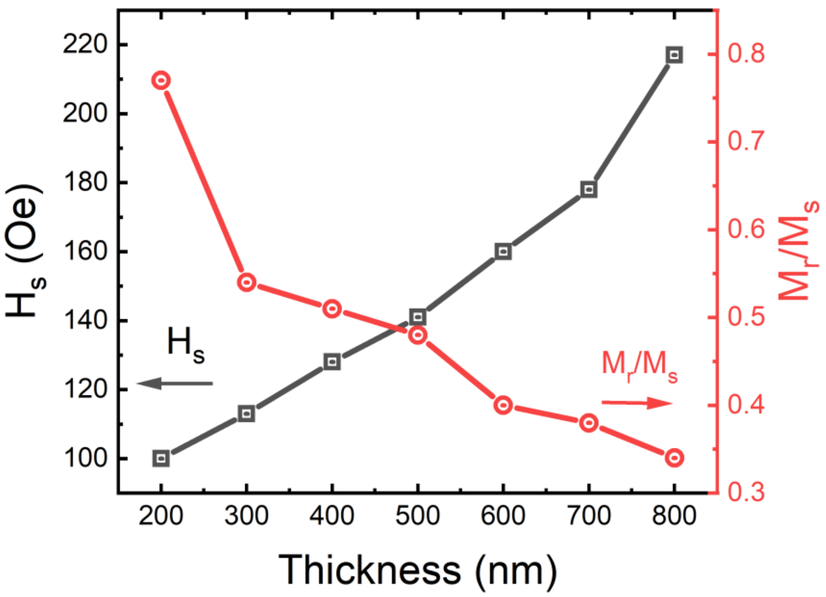

Supplementary Fig. 9. Normalized remanent magnetization (M_r/M_s, right axis) and saturation field (H_s, left axis) of NiFe films as a function of film thickness.

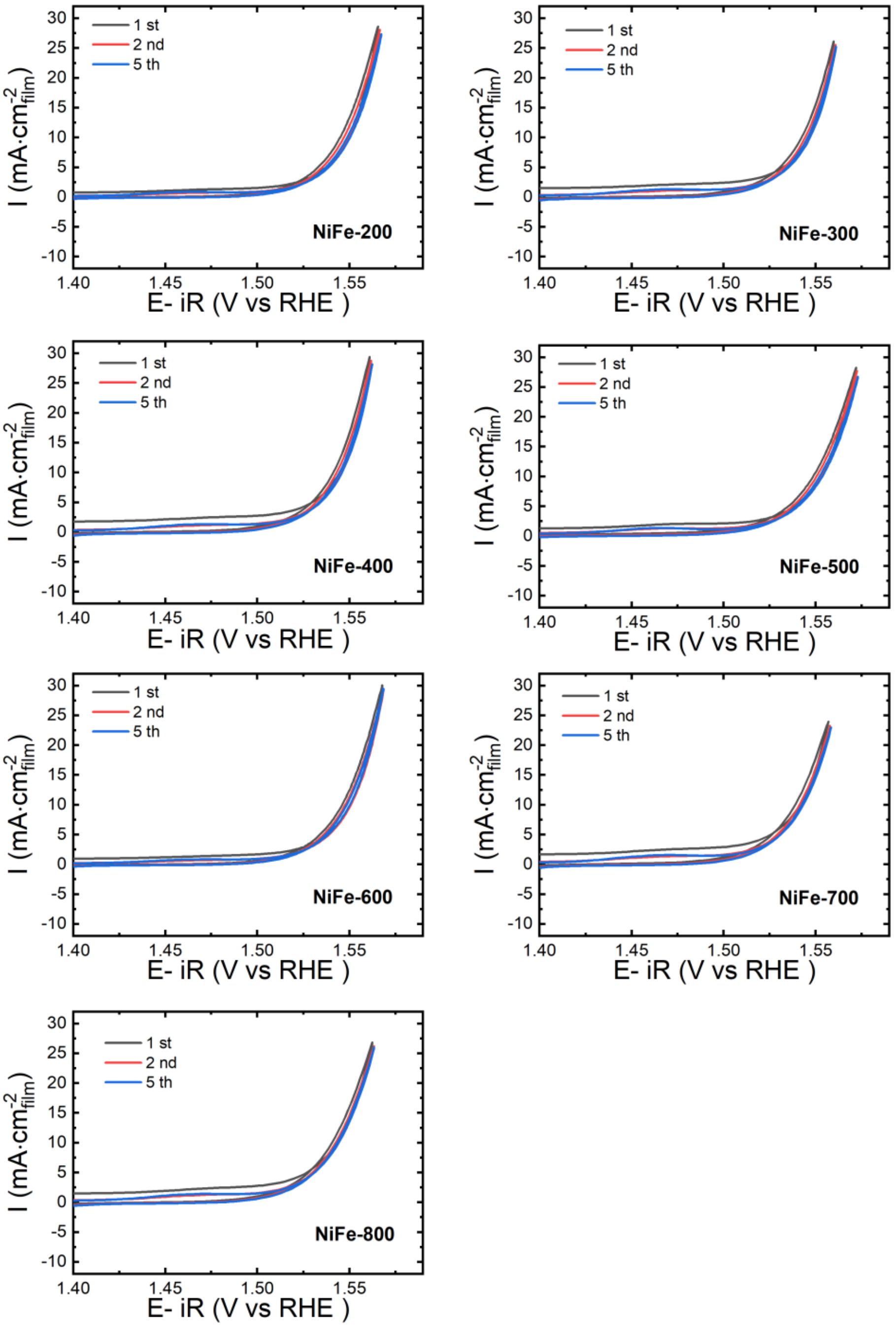

Supplementary Fig. 10. | Pretreatment for NiFe films. All NiFe were performed 5 CV cycles in the oxygen-saturated 1.0 M KOH at a scan rate of 100 mV s^{-1}. All films delivered stable CV profiles after 5 cycles.

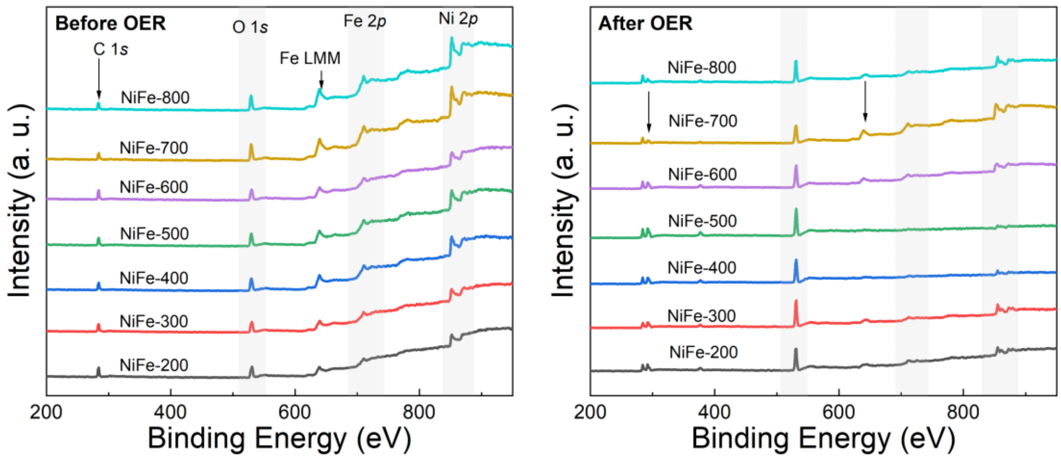

Supplementary Fig. 11. The XPS survey spectra of all film samples with and without the electrochemical treatment. The 1s orbital of O and the 2p orbital of Ni and Fe are labeled. Note that the broad peak appears above 600 eV which was identified as the Fe LMM peak. The electrochemically treated samples showed two peaks at C 1s, which may be contributed by carbonate[1]. The XPS survey spectra confirm the coexistence of Ni, Fe, and O in all film samples with and without the electrochemical treatment.

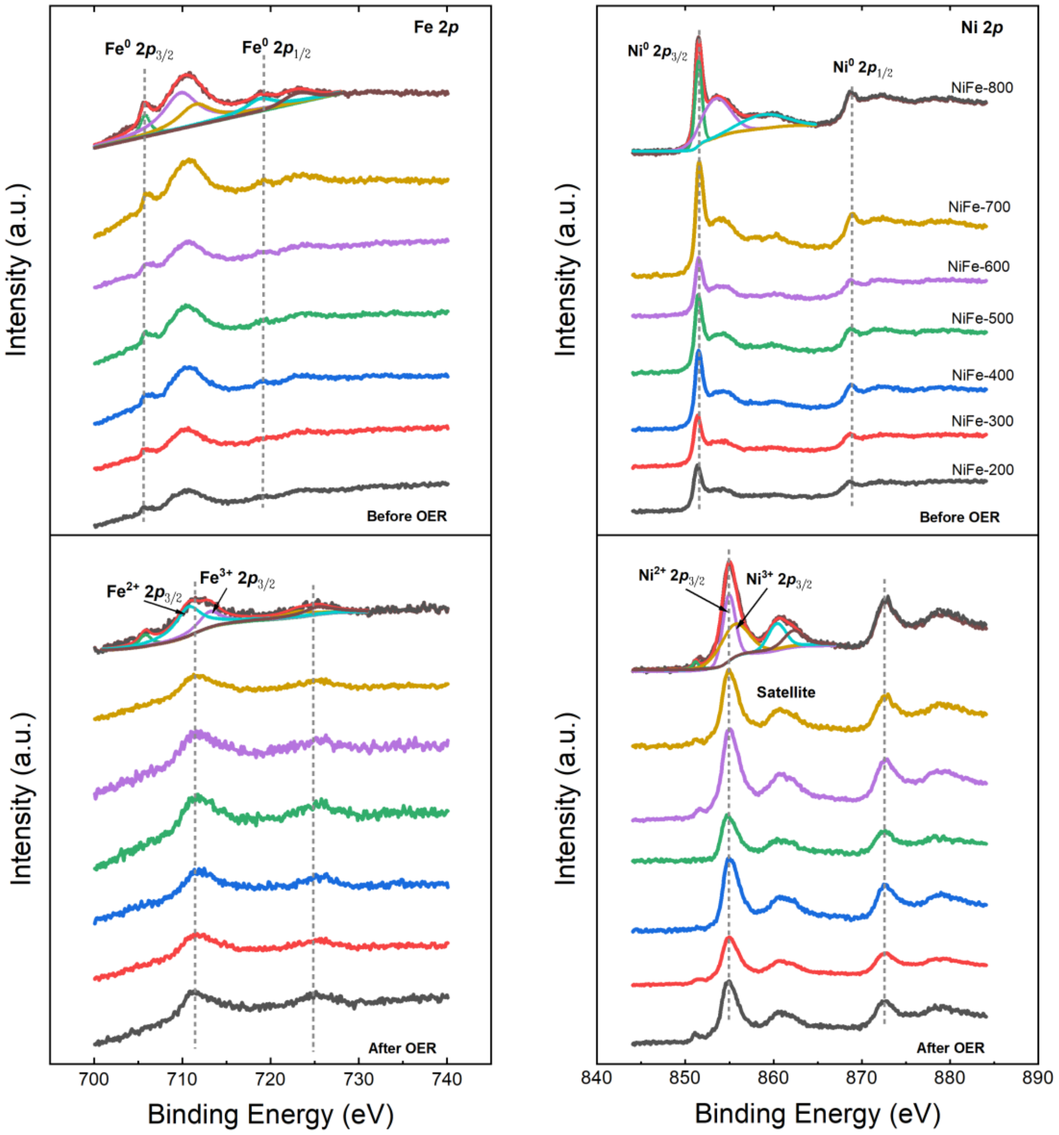

Supplementary Fig. 12. The Ni and Fe 2p for all NiFe films before and after the electrochemical tests.

As shown in Supplementary Fig. 12, the metallic Fe, Ni irons (labeled Fe^0 and Ni^0) with narrow peaks can be found in the initial film samples. The metallic Fe 2p core level presents two pairs of peaks: Fe^0 $2p_{3/2}$ at 706.2 eV and Fe^0 $2p_{1/2}$ at 719.2 eV[2]. The Ni 2p spectrum of Ni metal has its main peak near 852.5 eV and a second, comparatively low intensity at about 868.8 eV[3]. After the electrochemical tests, the peaks of Fe0 become weakened and the XPS spectra of Ni 2p3/2 can be fitted with two characteristic peaks at 855.2 and 856.2 eV, which are associated with the formation of $Ni(OH)_2$ and NiOOH, respectively [4]. The result indicates that the $NiFeO_xH_y$ is formed on the surface.

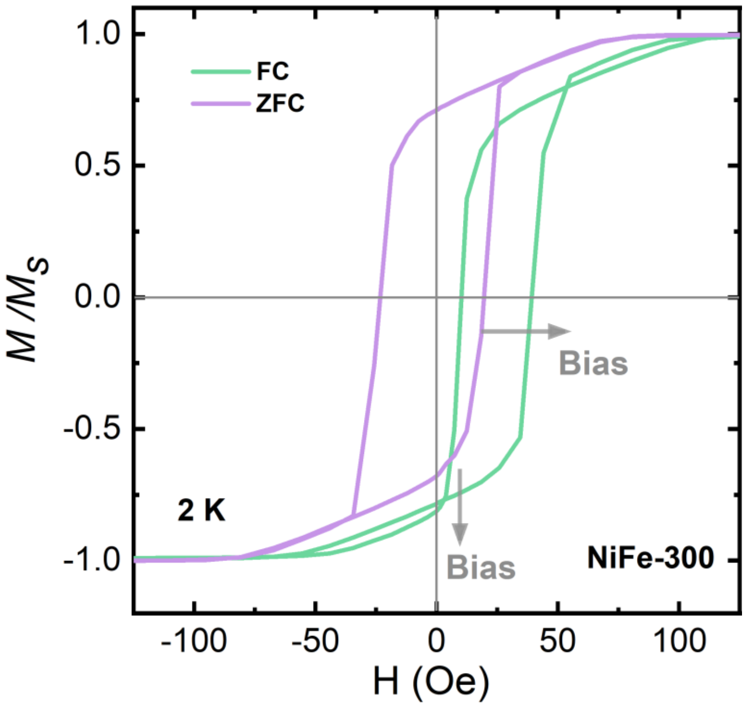

Supplementary Fig. 13. The magnetic hysteresis loops of NiFe-300 film after OER treatment (i.e., NiFe/Ni(Fe)O_xH_y) under both field cooled (FC) mode and zero-field-cooled (ZFC) mode at 2 K. The hysteresis loops show large coercivity (HC) and a notable magnetization switching behavior at around zero field under field-cooled (FC) mode and zero-field-cooled (ZFC) mode. The down and right shift of NiFe/NiFeO_xH_y under FC mode indicate an exchange bias effect that originates from uncompensated interfacial spins that are pinned in the oxyhydroxide layer and do not follow the external magnetic field[5].

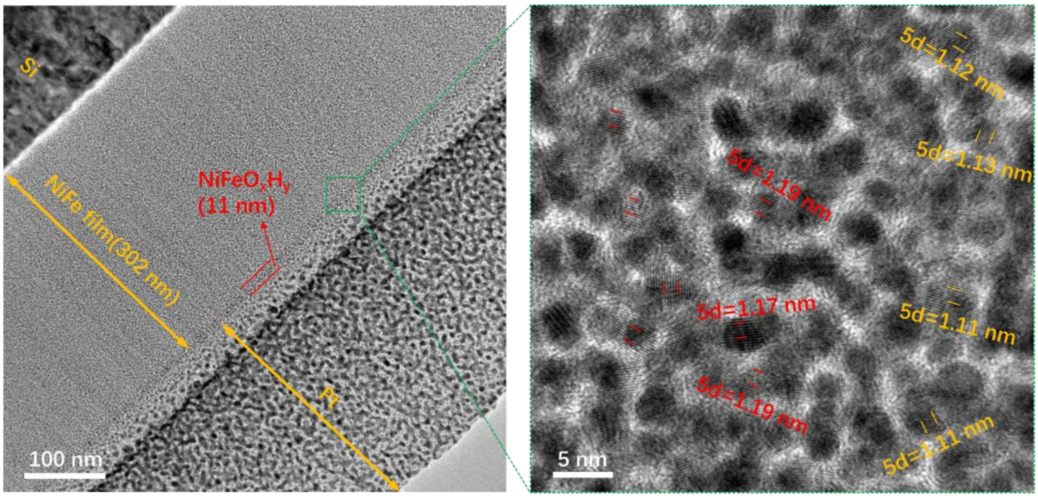

Supplementary Fig. 14. The TEM images of Si/NiFe/Pt film, where presents a multi-layer structure with distinctly different contrast. The right panel is zoomed in film surface, where nanoparticles emerge from the NiFe films under the electron beam illumination.

A transmission electron microscopy (JEM-2010Plus) was used to check the thickness of the surface layer in NiFe-300 after the electrochemical treatment. To protect the surface from focused ion beam (FIB) milling during specimen preparation, Pt was sputter-deposited at room temperature onto the sample. The architecture of Si/NiFe/Pt thin film can be clearly found in Supplementary Fig. 14. We here focus on the surface of the NiFe film, where presents a multi-layer structure with distinctly different contrast. The outermost layer is identified as Pt(111) lattice planes (PDF#04-0802). It is noted that the NiFe and $NiFeO_xH_y$ are in amorphous form. Nanoparticles (~2.5 nm) emerge from the NiFe films after a short-term electron beam illumination. Thus, we cannot distinguish between NiFe and $NiFeO_xH_y$ by lattice spacing. By the contrast difference, the thickness of the $NiFeO_xH_y$ surface layer can be estimated as about 11 nm.

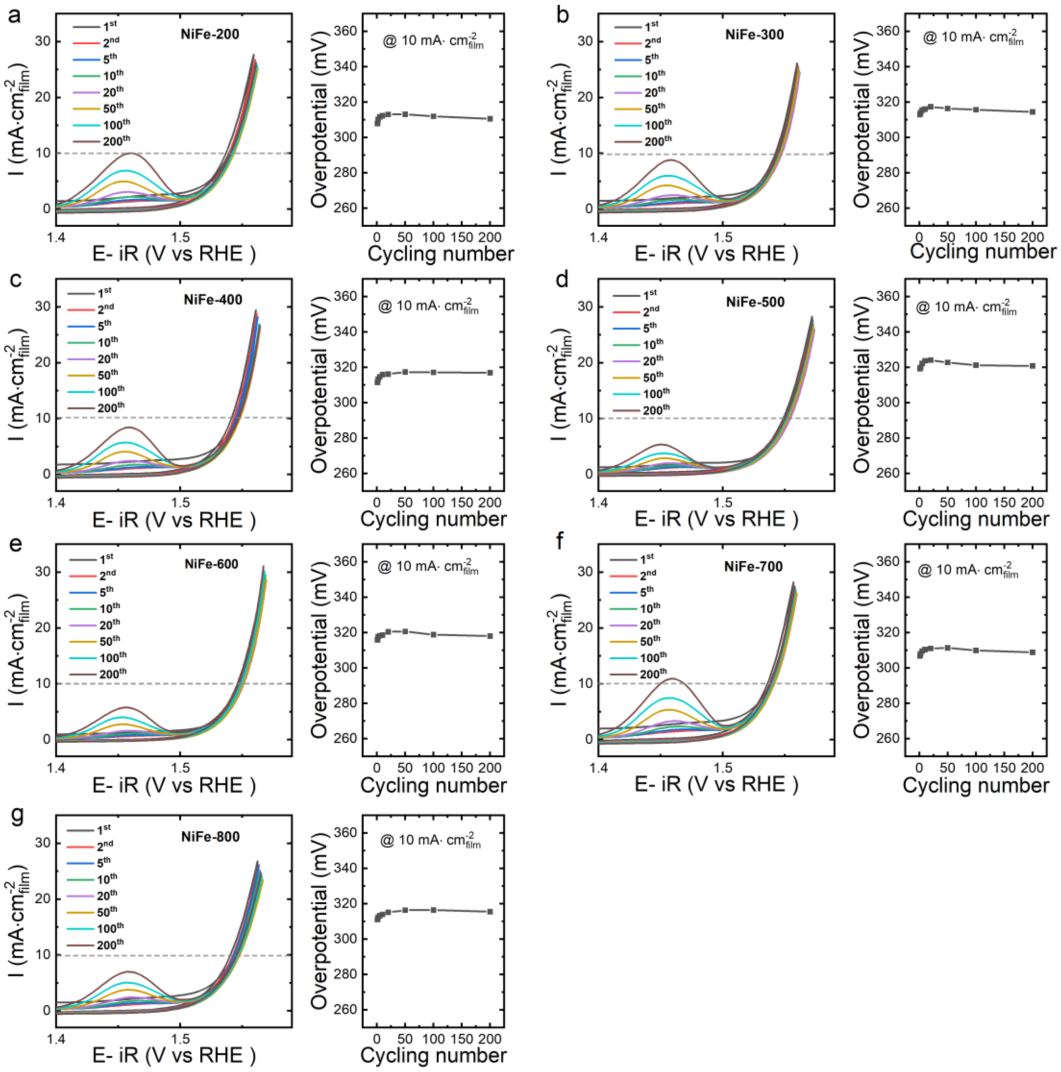

Supplementary Fig. 15. All NiFe electrodes were performed 200 CV cycles in the oxygen-saturated 1.0 M KOH at a scan rate of 100 mV·s^{-1}. Evolution of the overpotentials (η) during cycling at 10 mA·cm^{-2} extracted from the CV diagram show in right panel.

It is found that redox peaks of those NiFe films become obvious as the number of CVs increases, which indicates that more $NiFeO_xH_y$ species is formed on the surface. However, these films did not show significant changes in OER activity. After 100 CVs, the change in overpotential is almost negligible. The change of the overpotential at the 10 mA·cm^{-2} is within 1 mV between 100 CVs and 200 CVs. This small activity variation along with cycling is far below the activity enhancement under a magnetic field (similar to the situation when the measurement was conducted after 5 cycles). Thus, all of those films are pretreated after 100 CVs and then the experiments with the magnetic field are conducted.

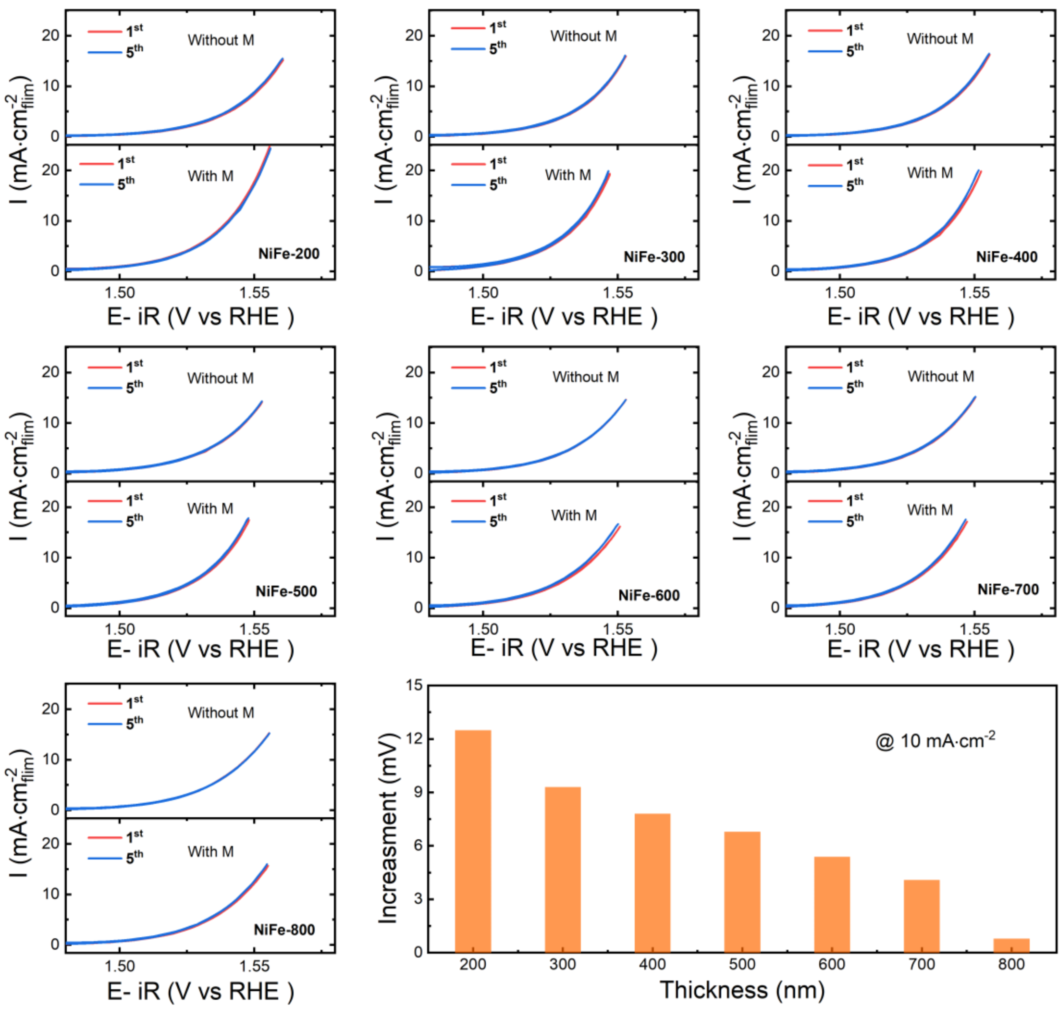

Supplementary Fig. 16. After 100 CVs Pretreatment, CVs of NiFe thin films with different thicknesses were performed at a scan rate of 10 mV·s^{-1} in O^{2-}saturated 1.0 M KOH with and without a constant magnetic field (2,000 Oe) for 5 circles. And the OER overpotential drop of NiFe films with the applied magnetic field is displayed in the lower right corner. The results are highly consistent with the results of the pretreatment 5 CVs.

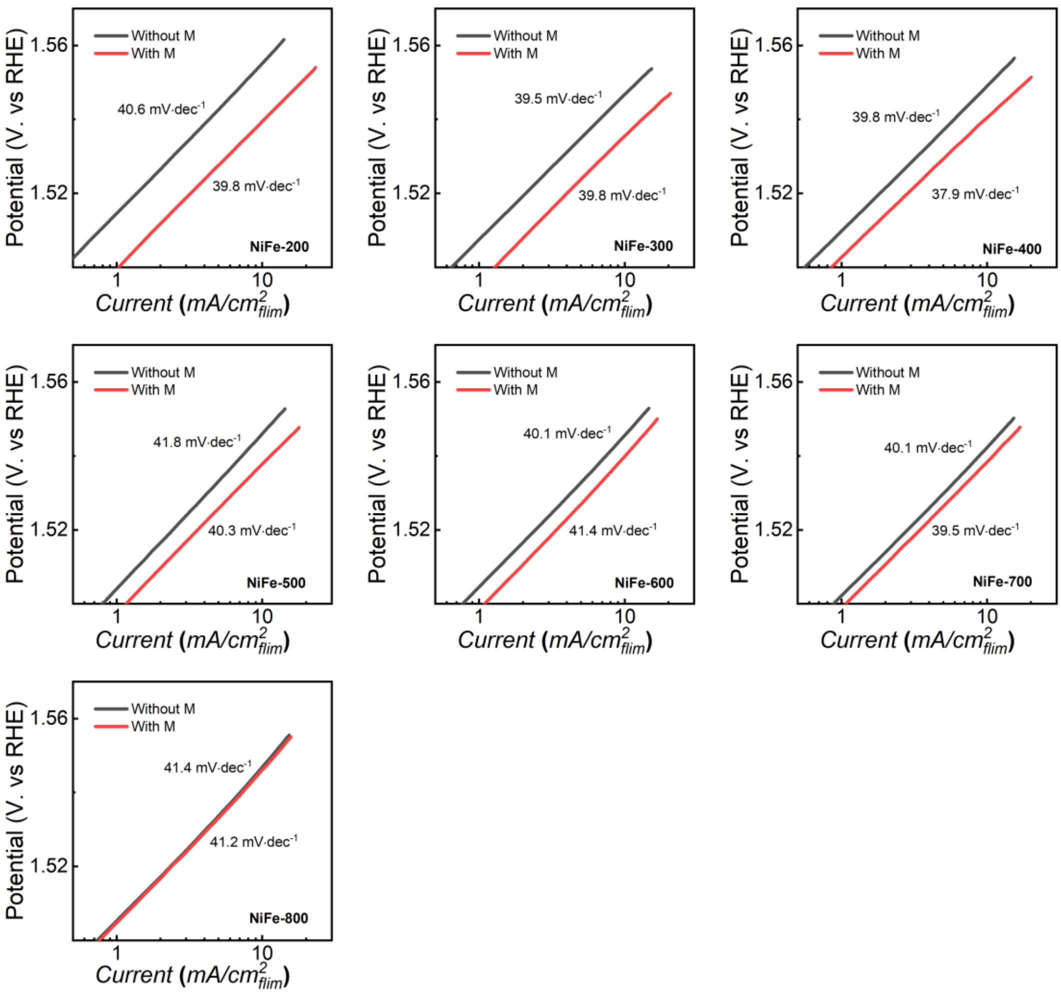

Supplementary Fig. 17. Tafel plot of NiFe thin films with different thicknesses with and without a constant magnetic field (2,000 Oe). The Tafel plots of NiFe films are shifted to lower potential with the magnetic field applied. The Tafel slope does not show remarkable change under magnetization.

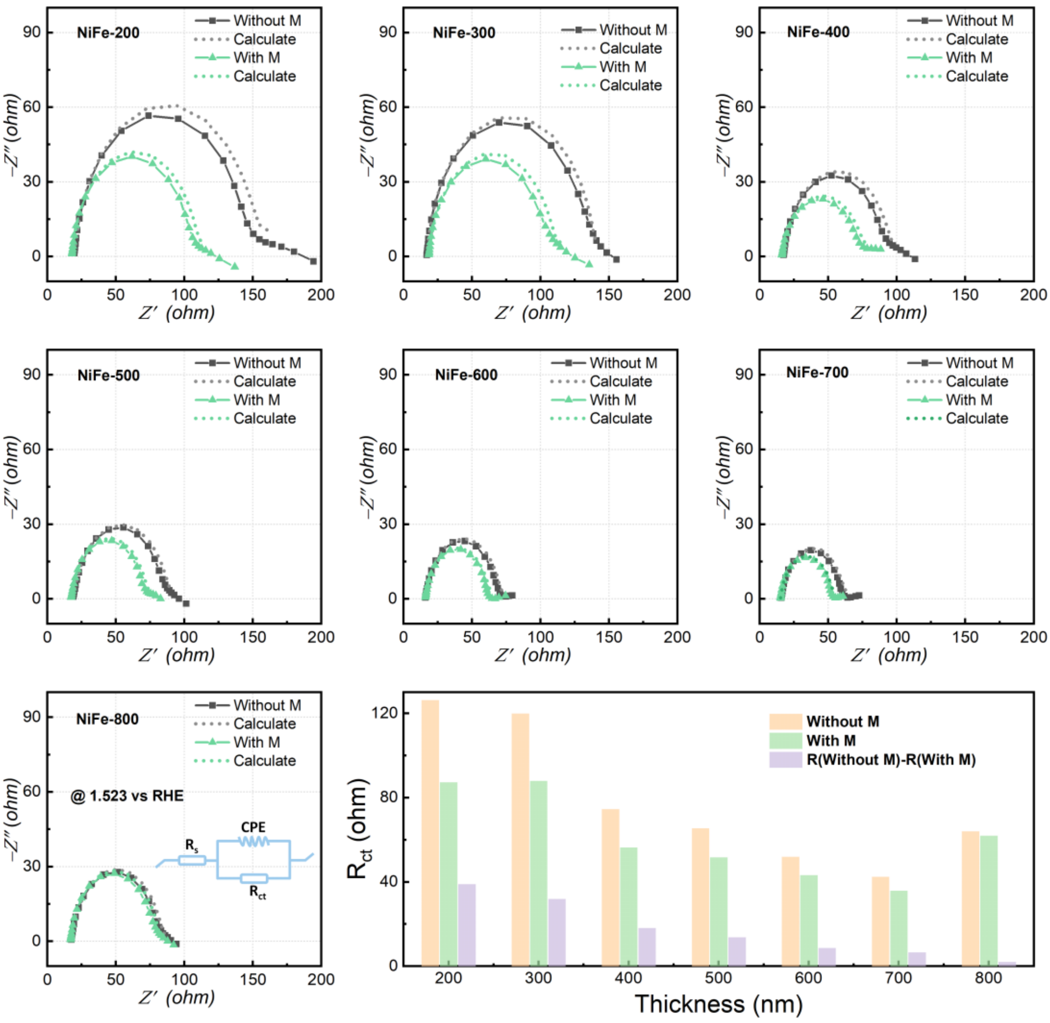

Supplementary Fig. 18. EIS of NiFe thin films with different thicknesses at 1.523 V versus RHE with and without a constant magnetic field (2,000 Oe). The EIS drop of NiFe films with the applied magnetic field is summarized in the panel at the lower right corner. The significant decrease in EIS of NiFe films applied in magnetic field that indicates faster electron transfer happens under the magnetization. It shows that the samples with a high domain wall ratio have faster electron transfer after magnetization, which is consistent with the OER enhancement.

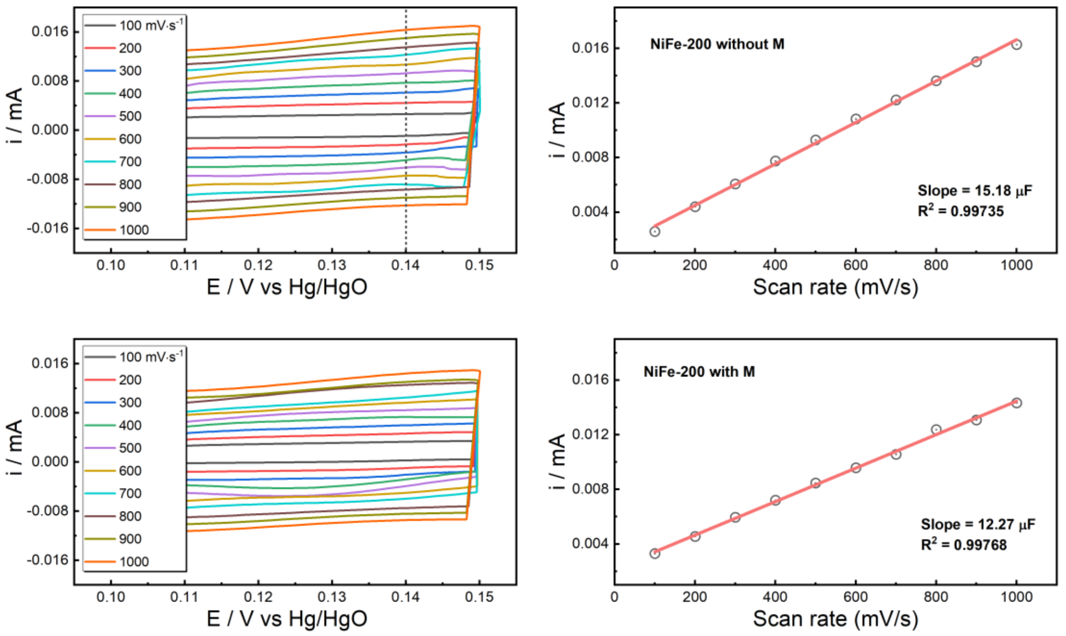

Supplementary Fig. 19. Double-layer capacitance measurements for NiFe-200 films with and without magnetic field (2000 Oe) from voltammetry in 1 M KOH. Left panel: CVs were measured in a non-Faradaic region of the voltammogram at various scan rates: 100 to 1000 mV· s^{-1}. The working electrode was held at each potential vertex for 10 s before beginning the next sweep. All current is assumed to be due to capacitive charging. Right panel: The anodic charging currents measured at 0.14 V vs Hg/HgO (1 M KOH) plotted as a function of scan rate. The determined double-layer capacitance of the system is taken as the absolute value of the slope of the linear fits to the data.

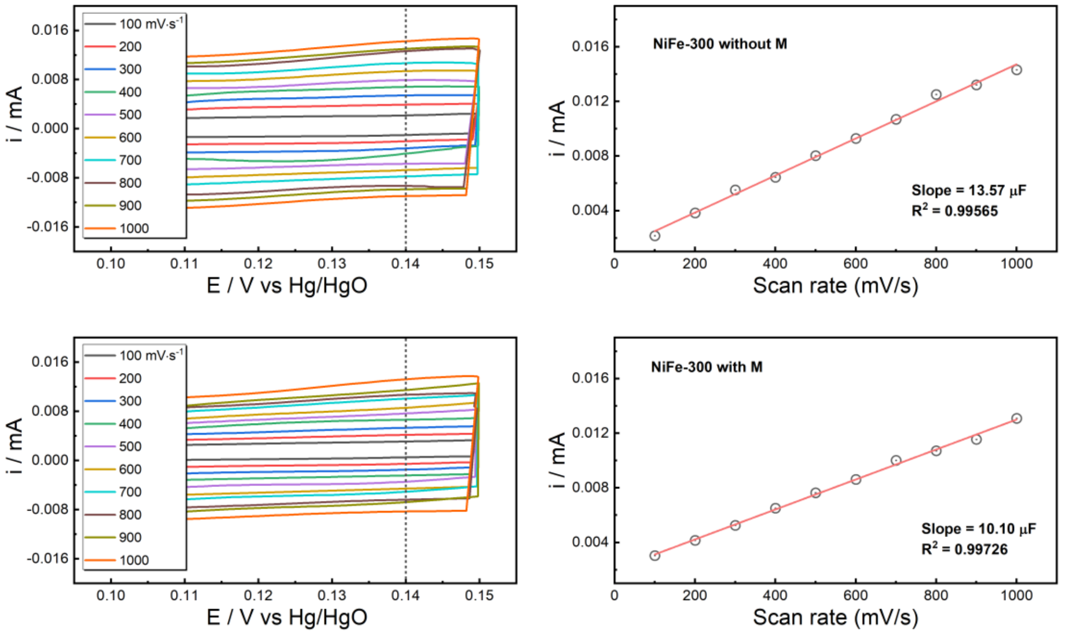

Supplementary Fig. 20. Double-layer capacitance measurements for NiFe-300 films with and without magnetic field (2000 Oe).

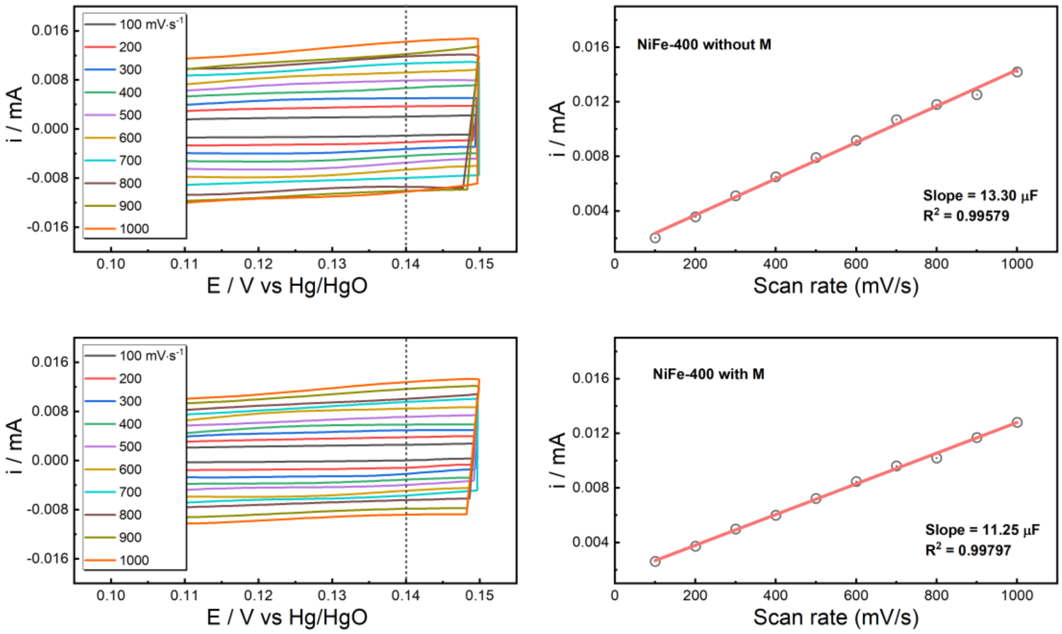

Supplementary Fig. 21. Double-layer capacitance measurements for NiFe-400 films with and without magnetic field (2000 Oe).

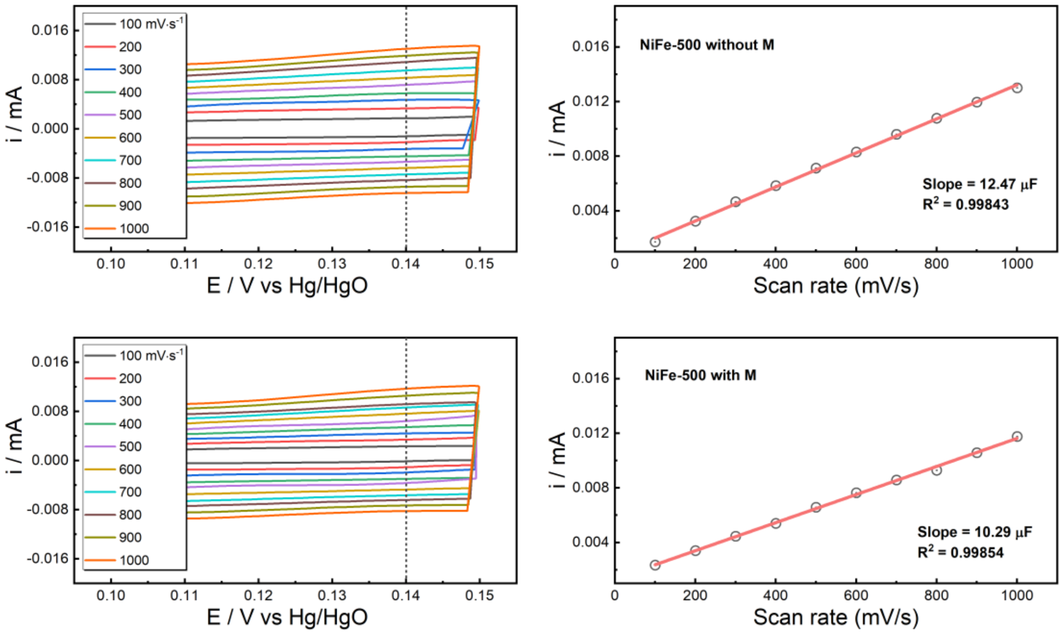

Supplementary Fig. 22. Double-layer capacitance measurements for NiFe-500 films with and without magnetic field (2000 Oe).

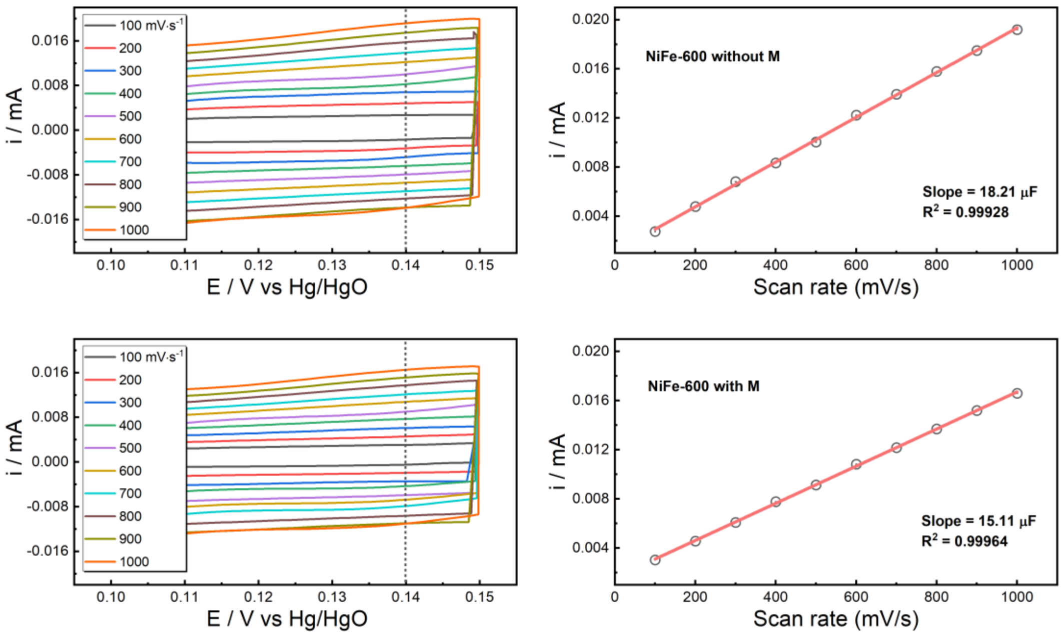

Supplementary Fig. 23. Double-layer capacitance measurements for NiFe-600 films with and without magnetic field (2000 Oe).

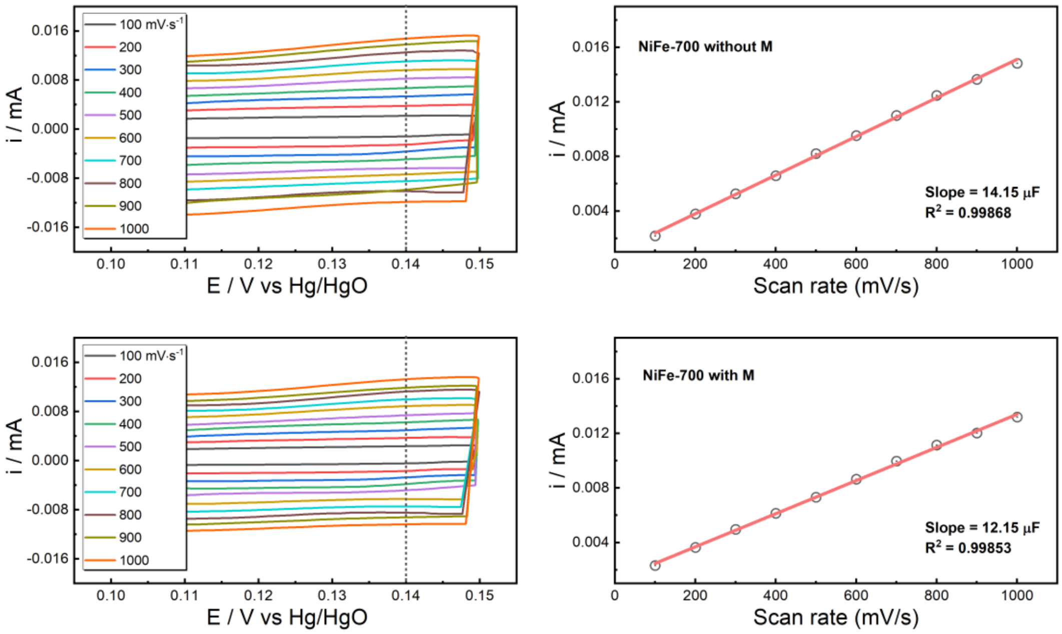

Supplementary Fig. 24. Double-layer capacitance measurements for NiFe-700 films with and without magnetic field (2000 Oe).

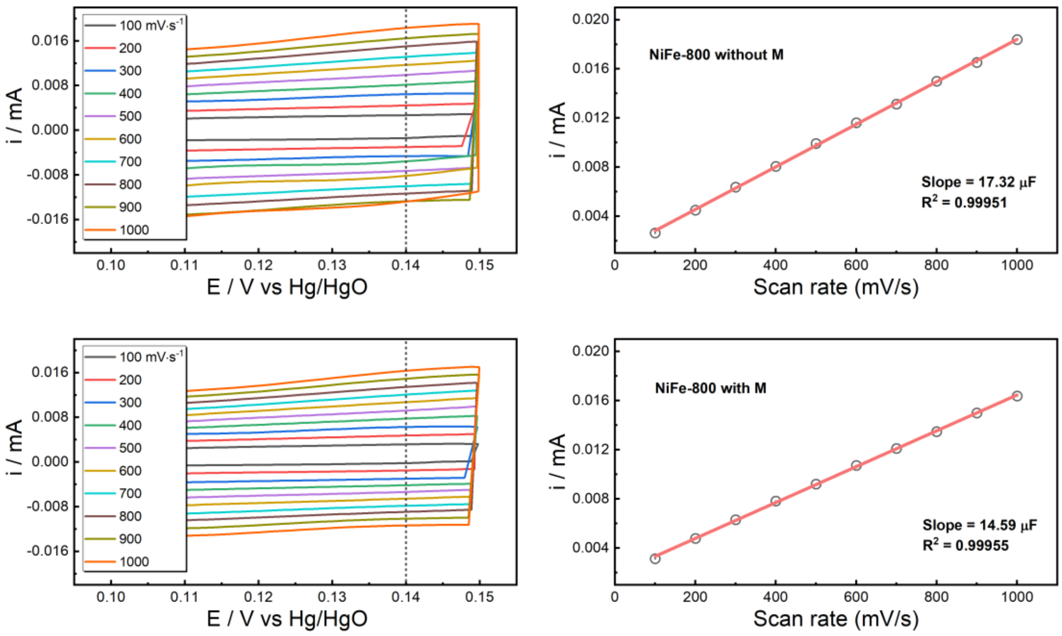

Supplementary Fig. 25. Double-layer capacitance measurements for NiFe-800 films with and without magnetic field (2000 Oe).

The double layer of NiFe films with and without magnetic field are shown in Supplementary Fig. 19-25. It is interesting that the double layer capacitance of all NiFe films is obviously reduced under magnetic field and this double layer capacitance drop has no thickness dependence. This is because that a tangential current flow is induced by the Lorentz force under the magnetic field, which leads to the charge of the double capacitor needing to move a longer distance at the same double layer thickness, thus exhibiting a smaller double capacitance. This effect is not related to the thickness of the films. Similar results have also been revealed in earlier works[6-9].

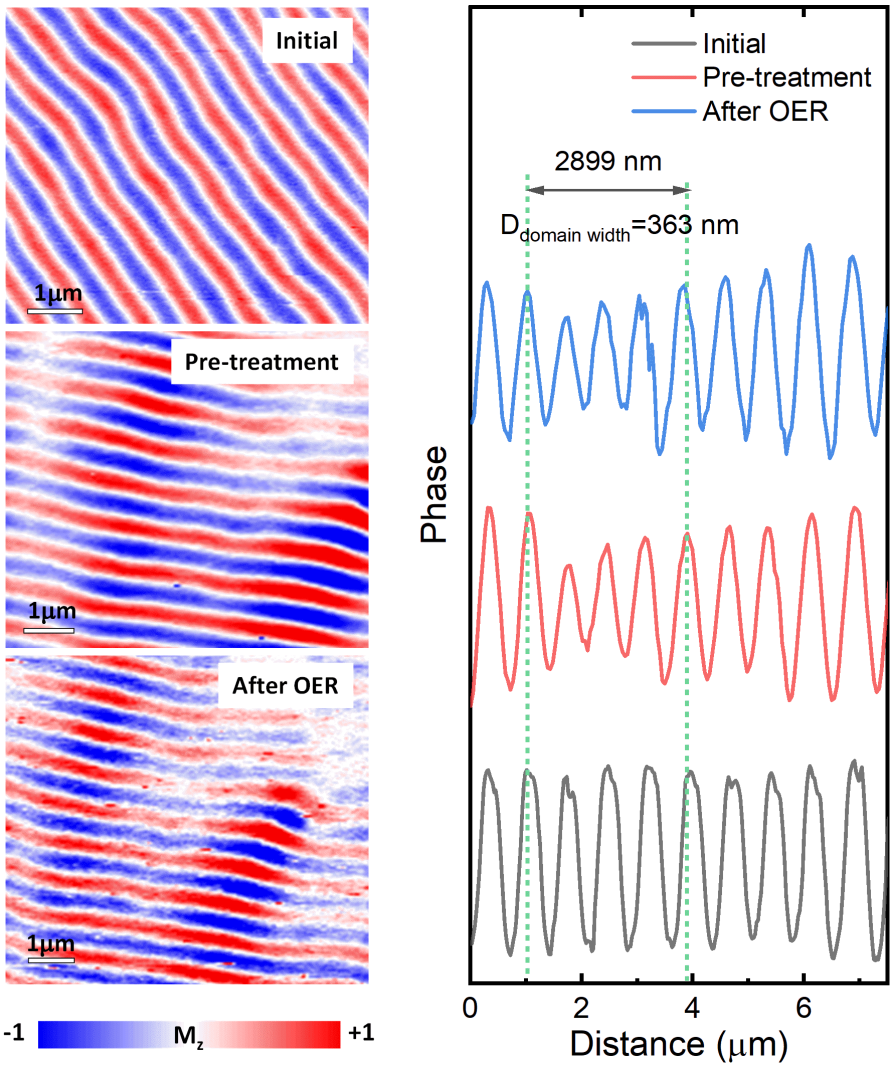

Supplementary Fig.26. Zero-field MFM images of the magnetic domain structure of NiFe-600 films for initial sample, after pre-treatment sample and after OER sample. The corresponding phase line profiles acquired from MFM images of NiFe films show in the right plane. The magnetic domain structures are all nearly the same in those stages, which indicates the electrochemical treatment has nearly no effect on the magnetic domain structure of these NiFe films.

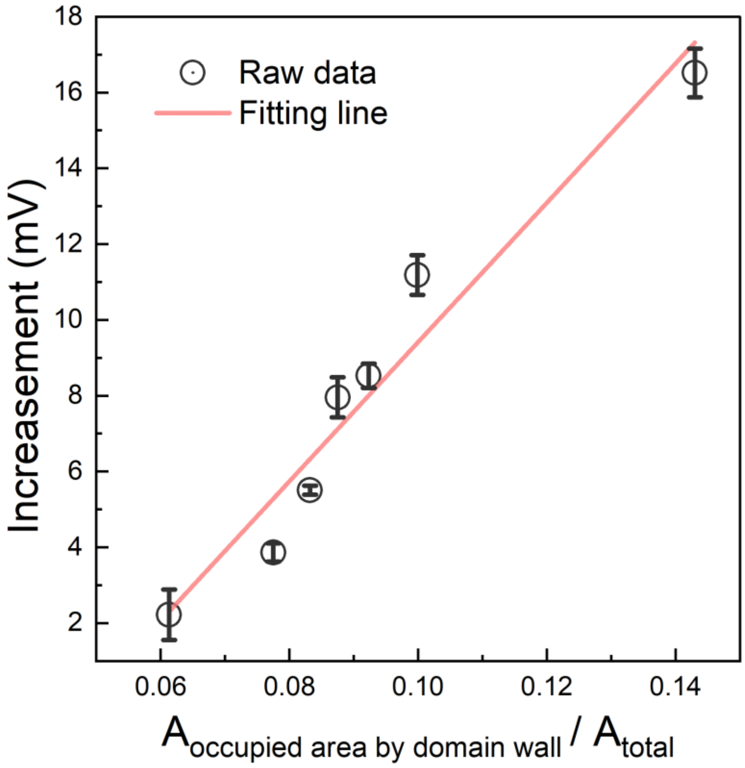

Supplementary Fig.27. The OER overpotential drop at 10 mA·cm_{film}^{-2} of NiFe films after magnetization as a function of the ratio of the surface area occupied by domain walls to the total surface area of NiFe films. Each data point represents three independent measurements.

We cation that the domain structure in surface of those NiFe films is more complex than bulk, where the magnetic moment has a mostly in-plane arrangement with a continuous rotation. But, the depth of this surface is about 4 nm based on micromagnetic simulations single cell size, which is much less deep than the depth of the catalyst film surface involved in the reaction (at least 11 nm according to TEM). More importantly, the OER enhancement is from the magnetization, the important information for comparison comes from the "Δ". The rate of change of the magnetic moment along the x-direction of the micromagnetic simulations surface shown in Supplementary Fig.28. The variation of magnetic moment is small in the domain region and large in the domain wall region. The rate of change of the magnetic moment at the surface corresponding to the domain wall shows a clear thickness dependence, i.e., the thicker the film and the wider the stripe, the smaller the rate of change of the magnetic moment. When the magnetic field is applied, all magnetic moment is pulled parallel, the thinner the sample, the greater the change in magnetic moment, which is consistent with the trend of OER enhancement.

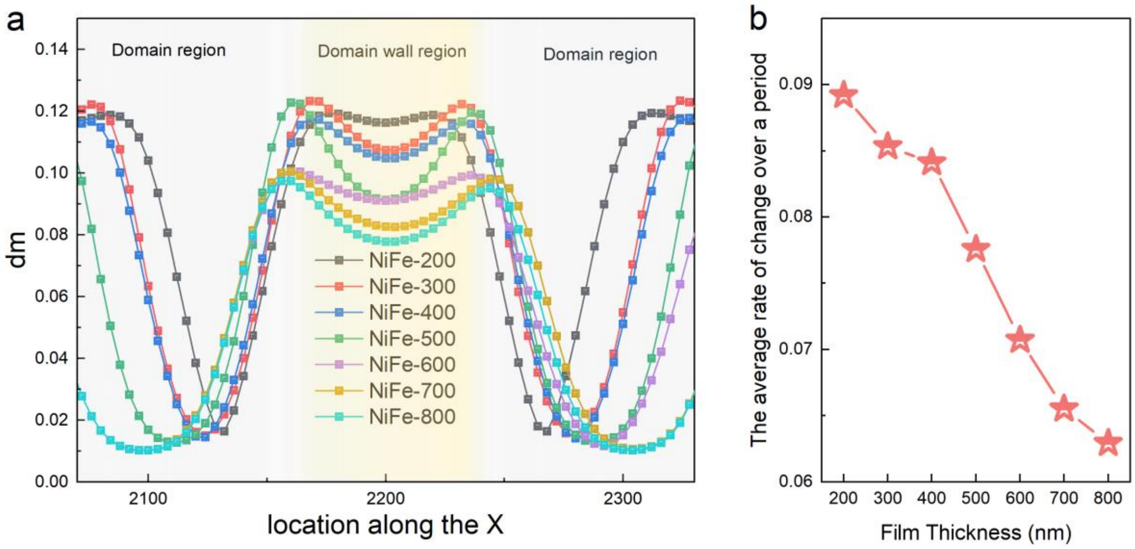

Supplementary Fig.28. **a.** The difference between two adjacent magnetic moments at different positions in the X-direction on the surfaces of samples with different thickness; **b.** The average rate of change of magnetic moment over a full stripe period.

Supplementary Table 1. | Film thicknesses and atom ratio obtained from SEM and EDX.

Samples	Real thickness /nm	Atom ration Fe:Ni
200	202	1:4.02
300	298	1:4.02
400	393	1:4.03
500	501	1:4.03
600	609	1:4.06
700	702	1:4.07
800	812	1:4.06

Supplementary Table 2. | Surface analysis of FeNi thin films obtained by atomic force microscopy (AFM). Parameters were extracted using IGOR pro. The film's area is given relative to a perfectly flat surface.

Samples	Domain width /nm	Roughness (Ra)/nm	Film's area	The area of domain wall ($\lvert M_z \rvert \leqslant 0.1$)
200	197	0.728	143.7%	0.143
300	257	0.785	146.1%	0.0999
400	278	0.861	141.2%	0.0923
500	352	1.108	137.9%	0.0875
600	366	1.094	131.9%	0.0832
700	405	0.941	137.8%	0.0775
800	438	0.863	143.7%	0.0613

Supplementary Table 3. | Summary of atomic ratio measurements.

Samples	Atom ration (Ni:Fe)		
	Region 1	Region 2	Region 3
NiFe-200	80.69%:19.31%	80.79%:19.21%	80.72%:19.28%
NiFe-300	80.89%:19.11%	80.68%:19.32%	80.47%:19.53%
NiFe-400	81.00%:19.00%	80.53%:19.47%	80.72%:19.28%
NiFe-500	80.87%:19.13%	80.97%:19.03%	80.65%:19.35%
NiFe-600	80.97%:19.03%	80.06%:19.94%	80.07%:19.93%
NiFe-700	80.52%:19.48%	80.87%:19.13%	80.64%:19.36%
NiFe-800	80.76%:19.24%	80.53%:19.47%	80.81%:19.19%

Supplementary Table 4. | The data of Tafel slope, double layer capacitance, and EIS for all NiFe films obtain from Supplementary Fig.17-25.

Samples	Tafel slope /mV·dec^{-1}		EIS/ohm			Double Layer (μF)		
	Without M	With M	Without M	With M	△	Without M	With M	△
200	40.6	39.8	126.3	87.35	38.95	15.18	12.27	2.91
300	39.5	39.8	119.9	87.92	31.98	13.57	10.10	3.47
400	39.8	37.9	74.56	56.34	18.22	13.30	11.25	2.05
500	41.8	40.3	65.44	51.7	13.74	12.47	10.29	2.18
600	40.1	41.4	51.91	43.25	8.66	18.21	15.11	3.10
700	40.1	39.5	42.45	35.9	6.55	14.15	12.15	2.00
800	41.4	41.2	64.02	61.94	2.08	17.32	14.59	2.73

References

1. Rouxhet, P.G. et al. XPS analysis of food products: toward chemical functions and molecular compounds. *Surf. Interface Anal.* **40**, 718-724 (2008).
2. Peter C.J. Graat, M.A.J.S. Simultaneous determination of composition and thickness of thin iron-oxide films from XPS Fe 2p spectra. *Appl. Surf. Sci.* **100** (1996).
3. Bancroft, H.W.N.á.D.L.á.G.M. Interpretation of Ni2p XPS spectra of Ni conductors and Ni insulators. *Phys Chem Minerals* **27**, 357-366 (2000).
4. Biesinger, M.C., Payne, B.P., Lau, L.W.M., Gerson, A. & Smart, R.S.C. X-ray photoelectron spectroscopic chemical state quantification of mixed nickel metal, oxide and hydroxide systems. *Surf. Interface Anal.* **41**, 324-332 (2009).
5. Nogués, J. & Schuller, I.K. Exchange bias. *J. Magn. Magn. Mater.* **192**, 203-232 (1999).
6. Olivier Devos, O.A., Jean-Paul Chopart, and Alain Olivier Is There a Magnetic Field Effect on Electrochemical Kinetics? *J. Phys. Chem. A* **104**, 1544-1548 (2000).
7. Monzon, L.M.A. & Coey, J.M.D. Magnetic fields in electrochemistry: The Lorentz force. A mini-review. *Electrochem. Commun.* **42**, 38-41 (2014).
8. Coey, J.M.D., Hinds, G., O'Reilly, C. & Ni Mhiochain, T.R. Magnetic Field Effects on Electrodeposition. *Mater. Sci. Forum* **373-376**, 1-8 (2001).
9. Oleg Lioubashevski, E.K., and Itamar Willner Magnetic Field Effects on Electrochemical Processes: A Theoretical Hydrodynamic Model. *J. Phys. Chem. B* **108**, 5778-5784 (2004).
